# Clinical Trials Portfolio and Regulatory History of Idelalisib in Indolent Non-Hodgkin Lymphoma: A Systematic Review and Meta-analysis

**DOI:** 10.1001/jamainternmed.2023.0190

**Published:** 2023-03-20

**Authors:** Titas Banerjee, Myung Sun Kim, Alyson Haslam, Vinay Prasad

**Affiliations:** 1 Division of Hematology and Medical Oncology, Knight Cancer Institute, Oregon Health and Science University, Portland; 2 Department of Epidemiology and Biostatistics, University of California, San Francisco

## Abstract

**Importance:**

Idelalisib is a first-in-class phosphatidylinositol 3-kinase inhibitor that received US Food and Drug Administration accelerated approval in July 2014 as a single-agent treatment for relapsed follicular lymphoma (FL) and small lymphocytic lymphoma (SLL). Serious adverse effects were reported in 2016 leading to termination of postmarketing registry trials. However, idelalisib remained on the market until 2022 when Gilead voluntarily withdrew the drug for the accelerated approval indication.

**Objective:**

Evaluate the regulatory oversight of the accelerated approval pathway and evidence generation for idelalisib during premarketing (2008-2014), postmarketing (2014-2016), and premarketing withdrawal periods (2016-2022).

**Data Sources:**

ClinicalTrials.gov, FDA.gov, PubMed database.

**Study Selection:**

Clinical trials investigating the safety and effectiveness of idelalisib.

**Data Extraction and Synthesis:**

Study characteristics and relative risk (RR) of safety outcomes were abstracted. Data were pooled using random effects meta-analysis. The analysis was performed in October of 2022.

**Main Outcomes and Measures:**

Trial status, recruitment status, publication status, serious adverse events (SAEs), fatal adverse events (FAEs), and all-cause mortality.

**Results:**

Overall, 31 idelalisib trials met selection criteria. In total, 20 of 30 (65%) included SLL and/or FL; 13 (42%) trials were completed, 13 (42%) had published results, and 7 (23%) were randomized clinical trials (RCTs). Overall, 6 RCTs of idelalisib had publicly available data on safety outcomes. By the initial postmarketing period (2016), the cumulative RR for SAEs was 1.86 (95% CI, 1.63-2.11), for FAEs was 3.30 (95% CI, 1.56-7.00), and for death was 1.35 (95% CI, 0.85-2.12). In the premarketing withdrawal period, only a single phase 3 trial was enrolling patients for FL and was terminated. However, idelalisib was not withdrawn from the market until 2022. Gilead reported cumulative sales revenue of $842 million during market authorization (2014-2022) and annual sales had a steady decline from $168 million to $62 million during the premarketing withdrawal period (2016-2021).

**Conclusions and Relevance:**

Findings of this systematic review and meta-analysis show that serious risks of SAE, FAE, and death with idelalisib treatment were evident by 2016. However, idelalisib remained on the market for another 6 years, with minimal evidence generation. It was voluntarily withdrawn for FL and SLL accelerated approval indications coinciding with decreasing revenue generation. Closer attention for safety and effectiveness of drugs reaching market by accelerated approval is needed.

## Key Points

**Question:**

What was the regulatory oversight and evidence generation for idelalisib between accelerated approval and voluntary withdrawal?

**Findings:**

In this systematic review and meta-analysis, we found 20 trials that investigated idelalisib in indolent non-Hodgkin lymphoma (NHL): 3 were phase 3 trials in indolent NHL, 2 of which were terminated due to increased mortality, and 1 recruited only 96 patients prior to voluntary withdrawal. No efficacy results were published for these trials; Gilead reported cumulative sales revenue of $842 million during market authorization.

**Meaning:**

Periodic assessment of regulatory effectiveness of the accelerated approval pathway is needed.

## Teaser

This systematic review with meta-analysis examines the regulatory oversight of the accelerated approval pathway and evidence generation for idelalisib during premarketing, postmarketing, and premarketing withdrawal periods.

## Introduction

Since the inception of the accelerated approval pathway in 1992, it has been an increasingly used strategy for drug approvals in oncology and hematologic malignant diseases. Between 1992 and 2017, 93 new oncology indications were approved under the accelerated pathway.^1,2^ Most accelerated approvals in oncology are based on surrogate end points, such as response rates (87%), and are required to do postmarketing requirements (PMR) studies to confirm clinical benefit. Completion of these requirements is often delayed. For example, of 93 accelerated approvals between 1992 and 2017, 37 (40%) had not completed PMR by 2018 and 5 (5%) drugs that received accelerated approval had been withdrawn.^1,2^

Several drugs in the phosphatidylinositol 3-kinase (PI3K) inhibitor class have received accelerated approval. The PI3K pathway has been of specific interest because of its central role in multiple cellular functions and signaling pathways in tumor development, and consequently, is a target for antitumor drugs.^3^ Idelalisib is a first-in-class PI3K inhibitor that received accelerated approval in July 2014 by the US Food and Drug Administration (FDA) as a single-agent treatment for relapsed follicular lymphoma (FL) and small lymphocytic lymphoma (SLL). Subsequently, additional PI3K inhibitors, including copanlisib, duvelisib, and umbralisib, were approved.^4^ However, during the first 2 years after approval, concerns about safety began to emerge, including risk of drug-related mortality.^4^ In 2022—7 years after the initial approval and more than 5 years after initial safety concerns were raised—Gilead Sciences, Inc voluntarily withdrew the idelalisib indications for FL and SLL, citing poor enrollment in confirmatory studies.^5^ This was followed by further withdrawals of next-in-class PI3K agents because further concerns were raised over the safety of the entire class of drugs.^6^

Idelalisib, a first-in-class drug, demonstrated early promising results and was granted accelerated approval, yet longer follow-up resulted in the manifestation of serious risks. Review of the regulatory history of idelalisib is instructive and offers lessons for improving oversight of the accelerated approval pathway. To address questions regarding the overall safety of idelalisib, the timing of its approval, and how decisions by both the FDA and Gilead Sciences coincided with the reporting and dissemination of safety data, we performed a cumulative meta-analysis of the evidence that emerged during market authorization and reviewed the regulatory response by the FDA. We focused on the sequence of events from accelerated approval to voluntary withdrawal of SLL and FL indications by Gilead Sciences and investigated the historical revenue generated by idelalisib within that time frame. We focused on 3 distinct time periods: the premarketing period (2008 to approval in 2014), initial postmarketing period (2014-2016, when safety concerns were raised), and premarketing withdrawal (2016 to withdrawal in 2022). We sought to understand what was known regarding the safety of idelalisib by the end of each of these 3 time periods by characterizing study designs, whether studies were completed, and evidence regarding safety, to determine whether its withdrawal could have occurred sooner. We also estimated the trajectory in revenue generated by sales of idelalisib over time.

## Methods

### Trials Data Set

A systematic review, including a cumulative meta-analysis, was performed by 1 reviewer (T.B.) for all clinical trials investigating the efficacy of idelalisib for indications that received regular or accelerated FDA approval. We searched ClinicalTrials.gov using the search term *idelalisib* and study type *interventional*, and PubMed with search term *idelalisib and lymphoma* and study type *clinical trials* to identify all idelalisib trials. Searches were from database inception through August 2022. All trials were reviewed following the Preferred Reporting Items for Systematic Reviews and Meta-analyses (PRISMA) reporting guidelines, and only trials with a clinical indication for chronic lymphocytic leukemia (CLL), SLL, or FL were included for analysis.^7^ For each trial, we abstracted the national clinical trial (NCT) number, title, start and end dates, phase, indication, trial status, and publication status. Studies listed as *active, not recruiting* were considered complete if results were published or shared on ClinicalTrials.gov, and the date on which the study status was changed from recruiting to not recruiting was noted instead of end date.

### Data Extraction for Randomized Clinical Trials

Analysis was performed on phase 3 randomized clinical trials (RCTs), excluding 1 extension study comparing 2 dose levels of idelalisib and 1 study comparing idelalisib to chlorambucil instead of placebo. From this set, we collected safety data reported on ClinicalTrials.gov. Data on the size of treatment and placebo groups, number of deaths, and number of serious adverse events (SAEs) for the placebo and treatment arms were collected. The number of SAEs was found under Study Results on ClinicalTrials.gov. Per their definition, SAEs are adverse events that are life-threatening or result in death, require an extend hospitalization, result in significant incapacity, or cause a congenital anomaly. All-cause mortality and rates of fatal adverse events (FAEs) not available on ClinicalTrials.gov were obtained from publications, if available. For trials that were terminated without reported results, data were collected from the FDA Briefing Document for the April 21, 2022, Oncologic Drugs Advisory Committee meeting discussing PI3K inhibitors.^8^ For all-cause mortality, the hazard ratio for death and confidence intervals (CIs) were obtained in addition to number of deaths.

### Statistical Analysis

Frequencies of trial characteristics such as phase, indication, status, and availability of published results were calculated. A timeline was created using the trial start and end dates for trials with SLL and FL indications. For the analysis of RCTs, cumulative risk ratios (RRs) of all-cause mortality, SAEs, and FAEs for the placebo and treatment arms were calculated based on the reported deaths, SAEs, and total participants. Using R statistical software (package meta, R Foundation), forest plots were then created from the calculated cumulative RRs for all-cause mortality, SAEs, and FAEs. The analysis was performed in October of 2022.

### Revenue Generated by Idelalisib

Worldwide sales revenue from idelalisib, reported by Gilead, was collected from the Securities and Exchange Commission (SEC) Annual Filings. We searched *Gilead* in the company search page of SEC.gov and reviewed the 10-K filings from 2014 to 2021 for reported worldwide idelalisib sales. The US-specific sales data were also collected when available.

In accordance with 45 CFR §46.102(f), this review was not human participants research and was not submitted to an institutional review board and did not require informed consent procedures.

## Results

### Characteristics of Idelalisib Trials

We identified 62 trials on ClinicalTrials.gov with search term *idelalisib* and study type *interventional*. An additional search of PubMed did not reveal additional studies. Of the 62 trials, 31 were excluded because they were for indications other than CLL, SLL, or FL, did not include idelalisib, were withdrawn prior to any patient recruitment, or were extension studies. The remaining 31 clinical trials were included in our analysis. Trial selection was notable for 31 trials in indolent non-Hodgkin lymphoma (NHL), 20 trials including SLL and/or FL, and 6 phase 3 RCTs (eFigure 1 in Supplement 1).

Among the 31 idelalisib trials for indolent NHL, we found that 11 (35%) were for CLL only but 20 (65%) included SLL and/or FL; the indications that have now been withdrawn. Most trials (23 [74%]) tested idelalisib in combination with other drugs.

Less than a quarter of studies were randomized trials (7 [23%]). Six (86%) of the RCTs compared idelalisib to placebo. Less than half (13 [42%]) of trials were completed and 13 (42%) had results published.^9,10,11,12,13,14^ Herein we analyze the characteristics and results of these trials in context of the regulatory history of idelalisib.

### Premarketing Period: June 2008 to July 2014

**Figure 1.**
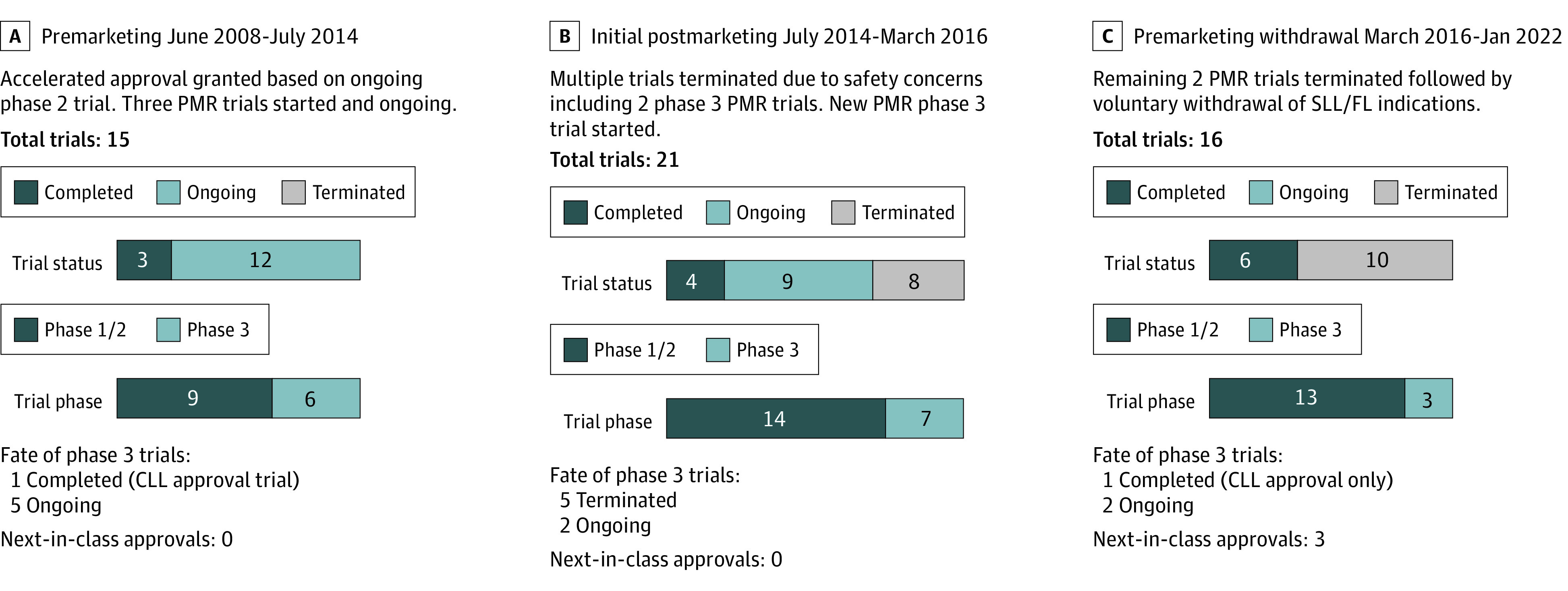
Summary of Idelalisib Trials and Regulatory History The salient features and findings of all 31 idelalisib trials with chronic lymphocytic leukemia (CLL), small lymphocytic leukemia (SLL), and follicular lymphoma (FL) indications are shown in context of the regulatory history of idelalisib. Data are grouped into 3 notable periods of time including the premarketing period, the initial postmarketing period, and the premarketing withdrawal period (after safety concerns were raised). Note that in each time period, total trials indicates all trials that were active during that period, and ongoing trials are those that continue into the next time period. Thus, some trials are counted in more than 1 period. Trials completing or terminating within 2 months after the time period are considered in the prior time period. PMR indicates postmarketing requirements.

**Figure 2.**
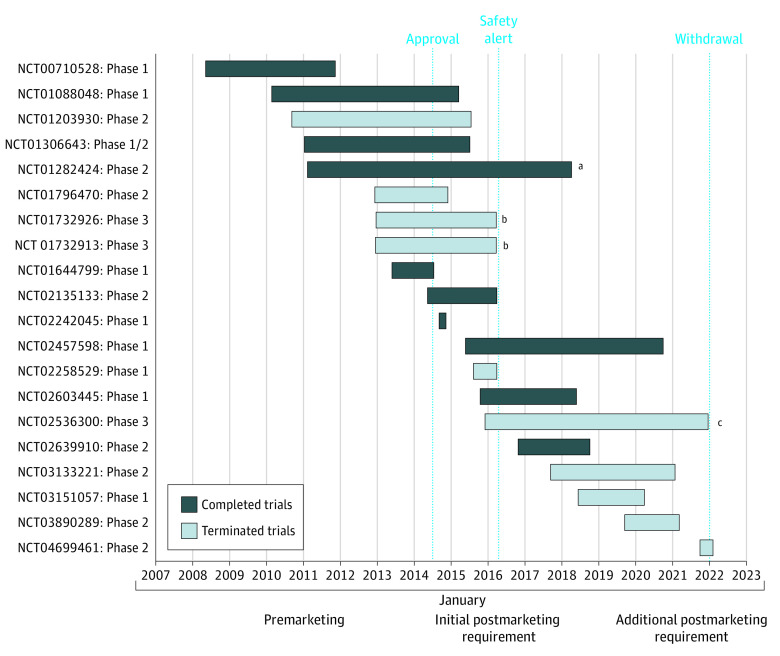
Timeline of SLL and FL Idelalisib Trials A chronological depiction of all idelalisib trials with small lymphocytic leukemia (SLL) and/or follicular lymphoma (FL) indications shown in relation to key dates including date of accelerated approval for SLL and FL indications, date when multiple trials were terminated due to safety concerns, and date of voluntary withdrawal of SLL and FL indications. The trial that led to accelerated approval and those that were required for regular approval are highlighted. ^a^Trial that led to accelerated approval. ^b^Initial postmarketing requirement. ^c^Additional postmarketing requirement.

Between June 2008 and July 2014, 15 trials studying idelalisib in CLL, SLL, and FL treatment were started. Most of these preapproval trials were early phase (phase 1 or 2) (9 of 15 [60%]) and 3 of 15 (20%) were completed (Figure 1). Most (9 of 15 [60%]) of these trials included SLL and/or FL (Figure 2). The completed trials included 1 phase 3 trial (NCT01539512), which, in July 2014, led to traditional approval of idelalisib in combination with rituximab for relapsed CLL.

**Figure 3.**
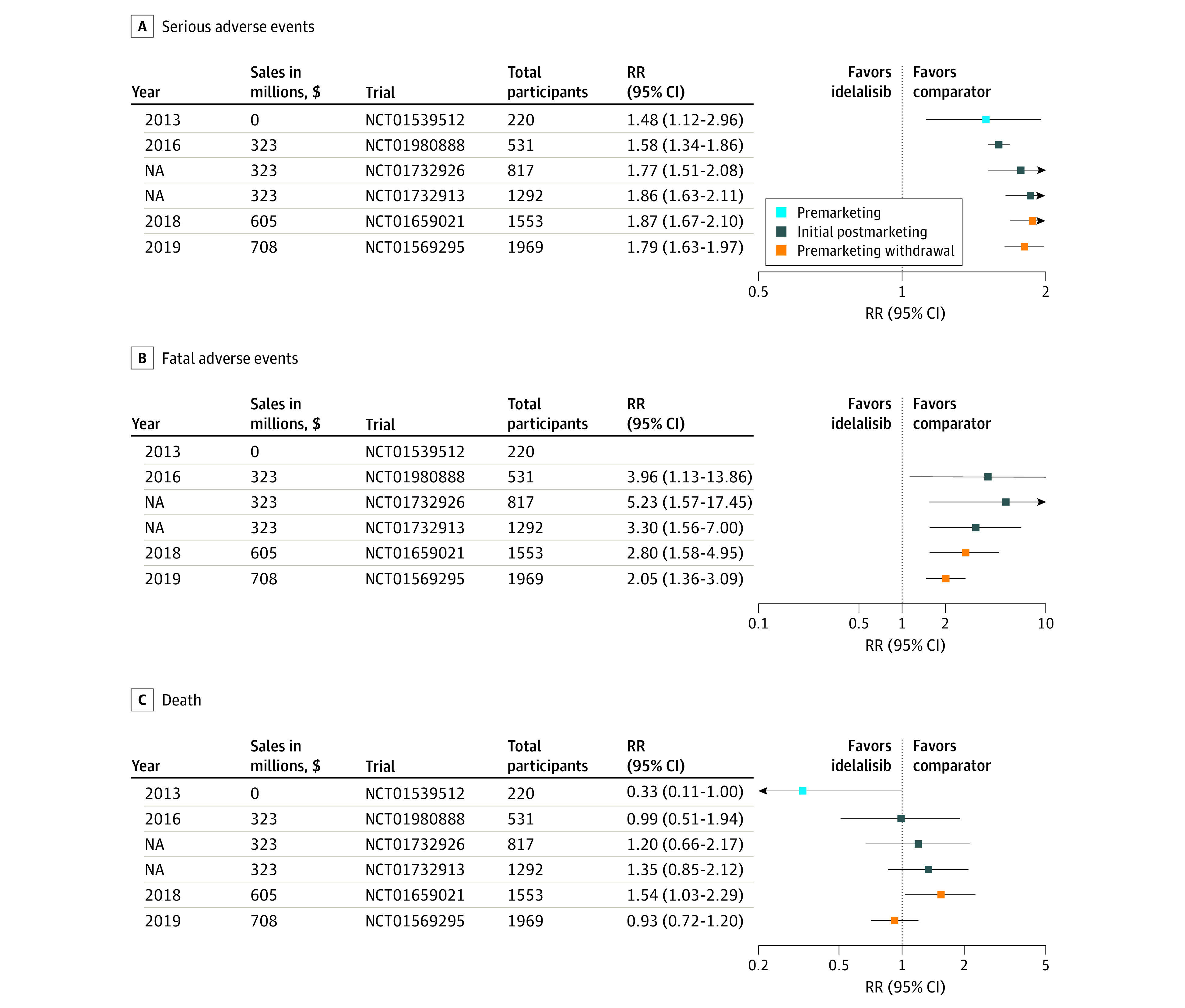
Forest Plots for Adverse Events and Death A, Forest plots of cumulative risk of serious adverse events; B, fatal adverse events; and C, death are shown for all idelalisib randomized clinical trials comparing idelalisib with placebo. Because this is a cumulative analysis, each data point includes results from the study listed to the left and all studies above. Gilead’s cumulative earnings from the drug are also shown. NA indicates not applicable; RR, risk ratio.

In July 2014, idelalisib was also granted accelerated approval by the FDA as a single -agent treatment for relapsed FL and SLL after 2 lines of therapy. The accelerated approvals for FL and SLL were based on the then-ongoing phase 2 DELTA trial (NCT01282424), which showed a 54% overall response rate (ORR; complete + partial response) in 72 patients with FL and a 58% ORR in 26 patients with SLL, a surrogate unvalidated end point.^12^ Continued approval was contingent on further confirmation of clinical benefit through PMR. The safety data of idelalisib monotherapy were based on 146 adults with indolent NHL who received idelalisib in single-arm clinical trials. From initial approval in 2014, idelalisib carried a boxed warning regarding potential for fatal and serious toxic effects including hepatic toxic effects, severe diarrhea, colitis, pneumonitis, infections, and intestinal perforation. In later studies, severe allergic and skin reactions were recorded as well as *Pneumocystis jirovecii* pneumonia and cytomegalovirus infections.^15,16^ Based on the 1 phase 3 trial at this point, there was an RR for SAEs of 1.48 (95% CI, 1.12-1.96) with idelalisib treatment but no increased risk of death (Figure 3).

The initial PMR to obtain regular approval included completion of (1) a dose optimization trial for stable disease by June 2019 (NCT01539291), (2) a phase 3 trial comparing rituximab with or without idelalisib (NCT01732913) by December 2017, and (3) a phase 3 trial comparing bendamustine and rituximab with or without idelalisib (NCT01732926) by February 2019. All of these trials were started and ongoing at the time of accelerated approval.

### Initial Postmarketing Period: July 2014 to March 2016

Between July 2014 and March 2016, 21 trials were started or ongoing. Most of these trials were early phase trials (14 [67%]) (Figure 1). Thirteen (62%) included SLL and/or FL indications (Figure 2). Seven of these 21 trials were phase 3 RCTs and 3 of these 7 (43%) included SLL and FL indications. The 3 phase 3 RCTs included 2 of the original PMR trials and 1 that was established later.

In March 2016, the FDA issued an alert indicating that Gilead was stopping 6 clinical trials because of an increased risk of adverse events.^17^ Two of these were PMR trials for the SLL and FL indications (NCT01732913 and NCT01732926), and 5 of the 7 ongoing phase 3 RCTs during this time period were terminated. By the end of 2016, there were 4 completed/terminated RCTs comparing idelalisib vs placebo in indolent NHL. Based on data from those 4 studies, the cumulative RR for SAEs was 1.86 (95% CI, 1.63-2.11) and the cumulative RR for FAEs was 3.30 (95% CI, 1.56-7.00). Moreover, the cumulative RR for death by the end of 2016 was 1.35 (95% CI, 0.85-2.12) (Figure 3).

The FDA responded to these findings in September 2016 in 3 ways. They issued a new safety alert, updated the boxed warning and safety information on the drug label, and also added a requirement for the sponsor to conduct an additional trial (ie, a new PMR). This new PMR was issued to conduct a trial establishing a safe and effective dosing regimen of idelalisib in patients with relapsed FL by April 2024. As a result, a phase 3 RCT (NCT02536300) began in 2016, which compared 3 different doses of idelalisib.

During this initial postmarketing time period, Gilead’s year-over-year annual sales of idelalisib reached a peak of $168 million in 2016, with cumulative sales of $323 million (eFigure 2 in Supplement 1), which included sales for CLL in addition to FL and SLL indications.

### Premarketing Withdrawal Period: March 2016 to January 2022

Between March 2016 and January 2022, 16 trials were in progress. Given the termination of 5 phase 3 trials in the prior time period, 13 trials (81%) during this period were still early-phase trials (Figure 1). Of the 16 trials that were ongoing during this time, 9 included SLL and FL indications and 4 of these trials were completed by 2022 (Figure 2). There was only 1 phase 3 trial for SLL and FL indications in progress during this time.

Two phase 3 studies initiated prior to idelalisib approval in 2014 continued enrollment after 2016 (NCT01659021, NCT01569295). These were CLL-only studies, 1 completed and 1 terminated, both with results published. Only 1 other phase 3 trial enrolled patients and generated new data after 2016. This was the dose optimization study of idelalisib in FL (NCT02536300), the new PMR established in 2016 following the termination of prior PMR trials.

This final PMR trial for dose optimization in FL was terminated in January 2022, after recruiting 96 patients. On January 14, 2022, Gilead Sciences, Inc, voluntarily withdrew the idelalisib indications for FL and SLL, citing poor enrollment to confirmatory studies. Since the initial accelerated approval based on a phase 2 trial, no phase 3 trials in patients with SLL and FL that compared idelalisib with placebo were completed, and only 2 dose finding trials were ongoing after 2016.

The cumulative RR for adverse events and mortality beyond 2016 was generated by trials in CLL only (Figure 3). By 2018 the cumulative RR for mortality was noted to be higher for patients treated with idelalisib (RR, 1.54; 95% CI, 1.03-2.29) though this increased risk was not noted by the addition of the final CLL trial in 2019. However, by 2019, the cumulative RR of SAEs was 1.79 (95% CI, 1.63-1.97) and the cumulative RR of FAEs was 2.05 (95% CI, 1.36-3.09). Despite these concerns for safety, additional PI3K inhibitors received approval, including copanlisib in September 2017, duvelisib in September 2018, and umbralisib in February 2021.

### Sales Revenue From Idelalisib Reported by Gilead

Despite reports of increased mortality and FAEs, idelalisib continued to generate sales. Cumulative sales revenue by 2021 was $842 million (eFigure 2 in Supplement 1). The year-over-year annual sales revenue showed a steady decline starting in 2016, from a peak of $168 million in 2016 to $62 million in 2021. The US-specific sales data were available for 2016 to 2021 and accounted for 47% of all sales.

## Discussion

More than 7 years after the initial approval, Gilead Sciences, Inc, voluntarily withdrew idelalisib for indications for FL and SLL in January 2022, citing poor enrollment in confirmatory studies. In this systematic review and cumulative meta-analysis of clinical trials, we showed that only a single phase 3 trial was enrolling patients for FL after 2016: NCT02536300, a PMR trial required by the FDA after the termination of 2 other registry trials for indolent NHL in 2016. This trial ended recruitment in January 2022 with only 96 enrolled participants, although Gilead revenue data suggest that many more patients continued to receive this drug outside of clinical trials through 2022. The low trial participation, yet increasing revenues for Gilead, are set against a backdrop of accumulating evidence of increased mortality and a voluntary withdrawal of FL and SLL indications.

Our investigation revealed 5 key findings about the clinical trials portfolio of idelalisib for the indications of CLL, FL, and SLL. First, we found that less than half of the 31 studies (7 [31%]) were RTCs. Second, 13 of all 31 trials (42%) had published results. Third, the limited phase 3 data available showed increasing RR of SAEs, FAEs, and death, notably including an increased RR for death of 1.35 (95% CI, 0.85-2.12) that was known by 2016 (Figure 3). Fourth, this product was withdrawn for select indications in the US market following a 6-year period of insufficient data generation. Only 96 patients were enrolled in the single active phase 3 trial during this time. Fifth, during the years of marketing authorization, the company earned nearly a billion dollars, with an estimated 47% being US sales.

Could the drug have been withdrawn sooner to protect patients from a dangerous drug? Our analysis shows that the bulk of data had been accumulated by the end of the initial postmarketing period in 2016. From 2016 to 2022, patients were exposed to the toxic effects of the product, and physicians did not have access to critical data such as incidence of death or SAEs in terminated trials, while the drugs continued to have marketing authorization. From initial approval, idelalisib carried a boxed warning regarding potential for fatal and serious toxic effects. In March 2016, the FDA issued an additional warning about increased rates of adverse events and deaths in trials combining idelalisib with other cancer treatments after 6 trials were halted due to toxic effects and deaths.^17^ The FDA set confirmatory requirements at initial accelerated approval in 2014 and again in 2016, but enforcement of these requirements was delayed. It was not until 2022 that the drug was voluntarily withdrawn for FL and SLL indications.

The withdrawal did not occur due to new data. In fact, there were few ongoing trials after 2016, by which point safety concerns had already been raised. The decision did coincide with a period of decreasing revenue for Gilead (eFigure 2 in Supplement 1). Idelalisib was a promising drug at the time of approval in 2014, with predicted sales of $1.2 billion per year by 2020.^18^ Gilead purchased Calistoga Pharmaceuticals, which included idelalisib in its portfolio, for $375 million in 2011. Although the drug did not reach the highest blockbuster status, remaining on the market was more lucrative than withdrawal.

The case of idelalisib has implications for the entire class. During the 6 years between the time when safety concerns were raised and the withdrawal of the SLL and FL indications, the FDA approved several next-in-class drugs, including copanlisib, duvelisib, and umbralisib. The FDA decision to approve next-in-class drugs with a known safety signal for the parent is concerning. After the withdrawal of idelalisib, the accelerated approval for duvelisib was withdrawn in April 2022 and the accelerated approvals for umbralisib were withdrawn in June 2022.

The findings of our study suggest that patients would benefit from regulatory reform and increased oversight of the accelerated approval pathway. The Accelerated Approval Integrity Act, introduced in March 2022, would help protect patient safety by including an automatic expiration of accelerated approval status within 1 year if postapproval studies are not completed by the deadline.^19^

### Limitations

This study had 2 important limitations. First, these results may not be generalizable to all drugs and disease entities benefiting from the accelerated approval process. Second, we used data that were publicly available and thus were dependent on the accuracy and completeness of these databases.

## Conclusions

Idelalisib was voluntarily withdrawn for FL and SLL accelerated approval indications at a time of decreasing revenue generation. Six years prior to withdrawal there were already serious toxic effects concerns, and minimal evidence generation to show patient benefits. Evaluation of the effectiveness and integrity of accelerated approval pathway would improve patient safety.

## Notes

Supplement 1. eFigure 1. Flowchart of Trial Selection Process for Analysis eFigure 2. Cumulative Gilead Earning for Zydelig (2014-2021)

Supplement 2. Data Sharing Statement
