# Supplementary material for: Clinical Trials Portfolio and Regulatory History of Idelalisib in Indolent Non-Hodgkin Lymphoma: A Systematic Review and Meta-analysis
Source: JAMA Intern Med. 2023 Mar 20;183(5):435–41. doi: 10.1001/jamainternmed.2023.0190 (PMC10028543; doi:10.1001/jamainternmed.2023.0190)
Supplement: Supplement 1. — eFigure 1. Flowchart of Trial Selection Process for Analysis eFigure 2. Cumulative Gilead Earning for Zydelig (2014-2021) [file jamainternmed-e230190-s001.pdf]

Supplemental Online Content

Banerjee T, Kim MS, Haslam A, Prasad V. Clinical trials portfolio and regulatory history of idelalisib in indolent non-Hodgkin lymphoma: a systematic review and meta-analysis. *JAMA Intern Med*. Published online March 20, 2023. doi:10.1001/jamainternmed.2023.0190

eFigure 1. Flowchart of Trial Selection Process for Analysis

eFigure 2. Cumulative Gilead Earning for Zydelig (2014-2021)

This supplemental material has been provided by the authors to give readers additional information about their work.

© 2023 Banerjee T, et al. *JAMA Intern Med.*

eFigure 1: Flowchart of Trial Selection Process for Analysis

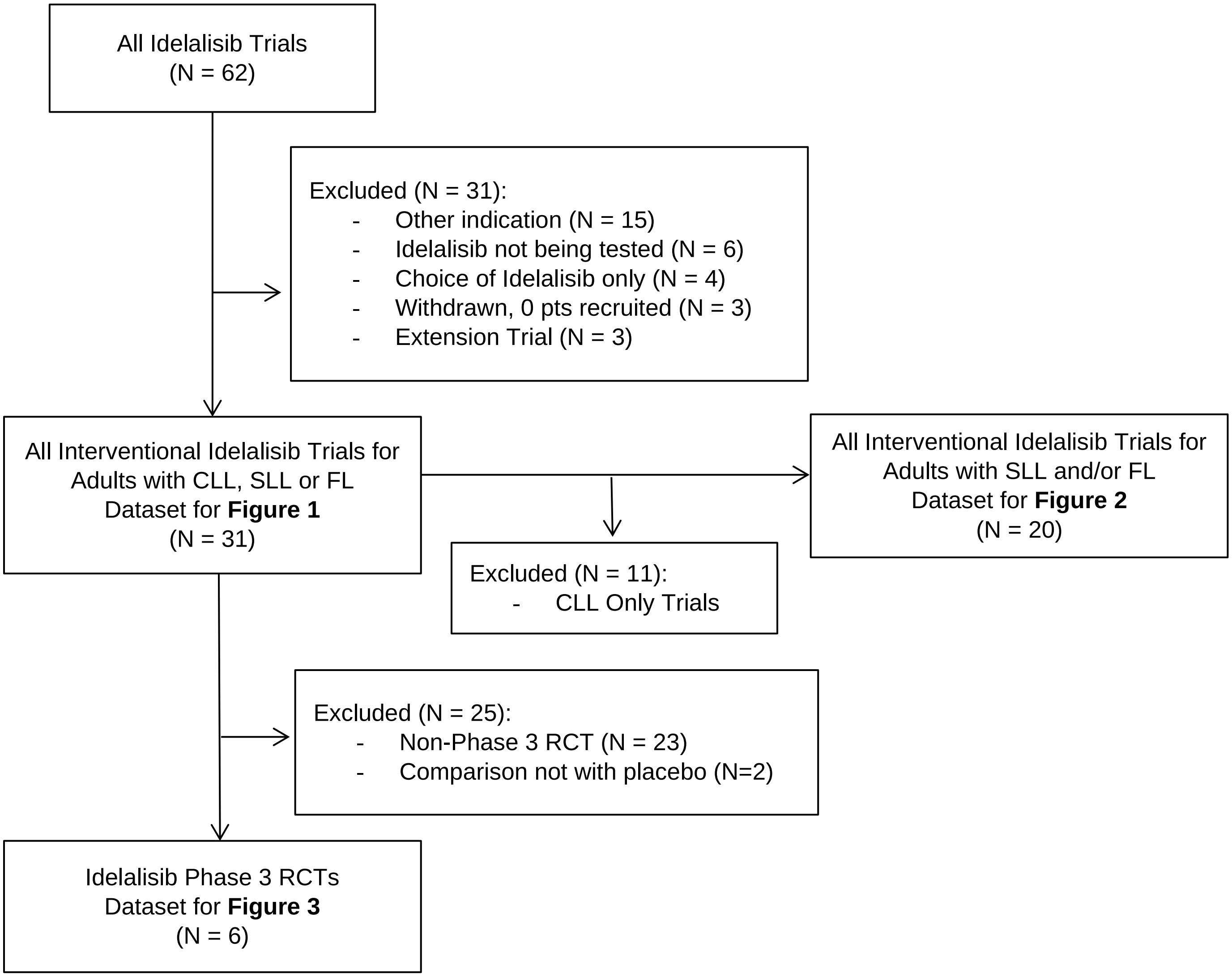

Overview of trial selection process for analysis

© 2023 Banerjee T, et al. *JAMA Intern Med.*

eFigure 2

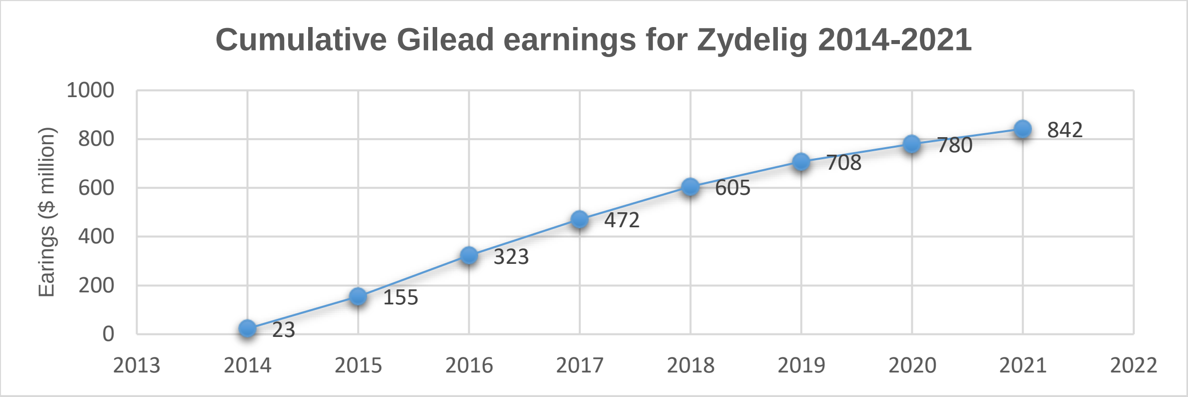

Cumulative Gilead Earning for Zydelig between approval in 2014 and voluntary withdrawal of SLL and FL indications in 2021

© 2023 Banerjee T, et al. *JAMA Intern Med.*.
