# Supplementary material for: Clinical Trials Portfolio and Regulatory History of Idelalisib in Indolent Non-Hodgkin Lymphoma: A Systematic Review and Meta-analysis
Source: JAMA Intern Med. 2023 Mar 20;183(5):435–41. doi: 10.1001/jamainternmed.2023.0190 (PMC10028543; doi:10.1001/jamainternmed.2023.0190)
Supplement: Supplement 2. — Data Sharing Statement [file jamainternmed-e230190-s002.pdf]

Data Sharing Statement

Banerjee. Clinical Trials Portfolio and Regulatory History of Idelalisib in Indolent Non-Hodgkin Lymphoma. *JAMA Intern Med*. Published March 20, 2023.
doi:10.1001/jamainternmed.2023.0190

Data
Data available: Yes
Data types: Data (not involving human participants)
How to access data: All study data is available upon request. Please email Titas Banerjee at banerjet@ohsu.edu
When available: With publication

Supporting Documents
Document types: None

Additional Information
Who can access the data: Anyone may request the data
Types of analyses: Data will be made available for any purpose
Mechanisms of data availability: The data will be made available via emailed file upon request
